# Effectiveness and safety of *Bifidobacterium* and berberine in human hyperglycemia and their regulatory effect on the gut microbiota: a multi-center, double-blind, randomized, parallel-controlled study

**DOI:** 10.1186/s13073-021-00942-7

**Published:** 2021-08-09

**Authors:** Jie Ming, Xinwen Yu, Xiaoqiang Xu, Li Wang, Chao Ding, Zhifeng Wang, Xuan Xie, Sheli Li, Wenjuan Yang, Shu Luo, Qingzhen He, Yafang Du, Zhufang Tian, Xiling Gao, Kaiyan Ma, Yujie Fang, Chen Li, Jiajun Zhao, Xiaokai Wang, Qiuhe Ji

**Affiliations:** 1 Endocrinology Research Center, Department of Endocrinology and Metabolism, Xijing Hospital, Fourth Military Medical University, Xi’an, 710032 China; 2 Aimigene Institute, Shenzhen, 518063 China; 3 Department of General Surgery, Nanjing Drum Tower Hospital, Medical School of Nanjing University, Nanjing, China; 4 Department of Endocrinology, The Second Affiliated Hospital of Xi’an Jiaotong University, Xi’an, China; 5 Department of Endocrinology, Affiliated Hospital of Yan’an University, Yan’an, China; 6 The Fifth Department of Internal Medicine, Shaanxi Aerospace Hospital, Xi’an, China; 7 Department of Endocrinology, Xi’an Daxing Hospital, Xi’an, China; 8 Genertec Universal Xi’an Aero-Engine Hospital, Xi’an, China; 9 Department of Endocrinology, Xi’an High-Tech Hospital, Xi’an, China; 10 Department of Endocrinology, Chang’an Hospital, Xi’an, China; 11 Department of Endocrinology, Xi’an Central Hospital, Xi’an, China; 12 Department of Endocrinology, Yan’an People’s Hospital, Yan’an, China; 13 Department of Endocrinology, Shangluo Central Hospital, Shangluo, China; 14 Department of Health Statistics, Fourth Military Medical University, Xi’an, China; 15 Department of Endocrinology and Metabolism, Shandong Provincial Hospital Affiliated to Shandong University, Jinan, 250021 China

**Keywords:** *Bifidobacterium*, Berberine, Gut microbiota, Hyperglycemia

## Abstract

**Background:**

Berberine and *Bifidobacterium* have been reported to improve glucose tolerance in people with hyperglycemia or other metabolic disorders. This study aimed to assess the hypoglycemic effect and the regulation of the gut microbiota caused by berberine and *Bifidobacterium* and the possible additive benefits of their combination.

**Methods:**

This was an 18-week, multi-center, randomized, double-blind, parallel-controlled study of patients newly diagnosed with hyperglycemia. After a 2-week run-in period, 300 participants were randomly assigned to the following four groups for 16 weeks of treatment: berberine (Be), *Bifidobacterium* (Bi), berberine and *Bifidobacterium* (BB), and placebo group. The primary efficacy endpoint was the absolute value of fasting plasma glucose (FPG) compared with baseline after 16 weeks of treatment.

**Results:**

Between October 2015 and April 2018, a total of 297 participants were included in the primary analysis. Significant reductions of FPG were observed in the Be and BB groups compared with the placebo group, with a least square (LS) mean difference of − 0.50, 95% CI [− 0.85, − 0.15] mmol/L, and − 0.55, 95% CI [− 0.91, − 0.20] mmol/L, respectively. The Be and BB groups also showed significant reductions in 2-h postprandial plasma glucose. A pronounced decrease in HbA1c occurred in the BB group compared to the placebo group. Moreover, compared with the Bi and placebo groups, the Be and BB groups had more changes in the gut microbiota from the baseline.

**Conclusions:**

Berberine could regulate the structure and function of the human gut microbiota, and *Bifidobacterium* has the potential to enhance the hypoglycemic effect of berberine. These findings provide new insights into the hypoglycemic potential of berberine and *Bifidobacterium.*

**Trial registration:**

ClinicalTrials.gov, NCT03330184. Retrospectively registered on 18 October 2017

**Supplementary Information:**

The online version contains supplementary material available at 10.1186/s13073-021-00942-7.

## Background

Type 2 diabetes (T2D) is one of the metabolic diseases with increasing prevalence worldwide [1]. Pharmacological intervention for T2D remains the gold treatment standard if lifestyle modification fails. Although various anti-diabetic drugs have been developed and used to treat diabetes, side effects and long-term efficacy pose frequent challenges [2]. Thus, safer and more effective medications are urgently needed. A series of studies suggest a link between the gut microbiota and human metabolic health. Mounting evidence has indicated that the gut microbiota affects the pharmacology of anti-diabetic drugs, and that in return, the metabolic products induced by these drugs transform the structure and function of the gut microbiota [3, 4]. Current studies have shown that the gut microbiota has become the target for anti-diabetic drugs.

Berberine is a major constituent of traditional Chinese medicine, *Coptis chinensis*, which is usually used as an antibiotic to treat diarrhea [5]. Existing studies have revealed its therapeutic effects on hyperglycemia and dyslipidemia in humans [6]. However, the mechanism of berberine is still unknown. Berberine administered orally has poor intestinal absorption and bioavailability [7] and low serum concentration [8], resulting in concerns over its effect on the gut microbiota. Previous animal studies have demonstrated that berberine may regulate the structure and function of the gut microbiota and alleviate insulin resistance by increasing the abundance of beneficial microbiota [9] such as *Bifidobacterium* [10], producing short-chain fatty acids [11], reducing the biosynthesis of branched-chain amino acids [12], altering the microbial bile acid metabolism [13], inhibiting the expression of intestinal inflammatory cytokines [14, 15], and improving the gut microbiota energy metabolism [16]. However, the evidence for the regulation of the human gut microbiota by berberine is limited. Some human studies reported the gastrointestinal reactions that occur after berberine treatment, including diarrhea and constipation, which may also be related to the gut microbiota [17–19]. In addition, the gut microbiota could enhance the biological activity of berberine by converting it into a more absorbable form, dihydroberberine [20]. Therefore, we hypothesized that the concomitant use of probiotics with berberine may improve the treatment effect.

*Bifidobacterium* is a widely used probiotic supplement. Population studies have indicated that oral supplementation of probiotics including *Bifidobacterium* could improve metabolic disorders such as T2D [21], gestational diabetes [22], excessive weight gain or obesity [23], metabolic syndrome, and non-alcoholic fatty liver [24, 25]. Berberine and the common anti-diabetic drugs acarbose and metformin have been shown to increase the abundance of *Bifidobacterium* after treatment [3, 4, 10]. However, due to the lack of studies about *Bifidobacterium* used alone, its effect on humans remains unclear. It is also not known whether *Bifidobacterium* can be fostered by other anti-diabetic medicines used for glucose control or whether the underlying mechanism involves regulating the intestinal environment to achieve a better hypoglycemic effect.

Therefore, this study was conducted to observe the hypoglycemic effect of berberine and *Bifidobacterium*, to verify the potential intestinal mechanism of berberine and *Bifidobacterium*, and to discern the possible benefits of adding *Bifidobacterium* to a berberine regimen.

## Methods

### Study design

The design of this study has been previously published, as a full study protocol [26]. Briefly, we performed a multi-center, randomized, double-blind, parallel-controlled study on newly diagnosed patients with hyperglycemia, which included a run-in period of 2 weeks and a treatment period of 16 weeks. This multi-center study was conducted in 10 tier 2 or 3 hospitals in Shaanxi province, China, between October 2015 and April 2018, and was approved by the independent Ethics Committee or institutional review board at each hospital. All of the participants provided written informed consent before study entry. This study was registered with ClinicalTrials.gov, NCT03330184.

### Participants

This study enrolled individuals aged 18-70 years with hyperglycemia, diagnosed by oral glucose tolerance test (the V1 stage was defined by fasting plasma glucose (FPG) of 5.6 ≤ FPG < 8.0 mmol/L, while the V2 stage was defined by 6.1 ≤ FPG < 8.0 mmol/L or a 2-hour postprandial plasma glucose [2-hr PPG] of 7.8 ≤ 2-hr PPG < 17 mmol/L at each sub-center laboratory), with a body mass index (BMI) of 19–30 kg/m^2^. Participants were excluded if they had participated in any other clinical trial within the prior 3 months. Individuals were also excluded if they met one or more of the following criteria: (1) had type 1 diabetes mellitus; (2) were diabetic and had previously treated or untreated FPG ≥ 8 mmol/L or 2-hr PPG ≥ 17 mmol/L; (3) were women of childbearing age who were pregnant, breastfeeding, or intended to become pregnant or were not using adequate contraceptive methods; (4) were allergic to the study drugs; (5) were unable to cooperate; (6) had impaired liver function, defined as an aspartate aminotransferase or alanine transaminase more than twice the upper limit of normal; (7) had impaired renal function, defined as serum creatinine ≥ 133 μmol/L; (8) had uncontrolled treated/untreated severe hypertension (systolic blood pressure ≥ 160 mmHg and/or diastolic blood pressure ≥ 95 mmHg); (9) had any chronic gastrointestinal disease (pancreatitis, inflammatory bowel disease) or history of intestinal surgery; (10) had severe heart disease, such as heart failure, unstable angina pectoris, acute myocardial infarction; (11) had chronic hypoxic diseases such as emphysema or pulmonary heart disease; (12) had obvious diseases of the blood system; (13) had tumor diseases or endocrine diseases, such as hyperthyroidism or hypercortisolism; (14) had mental illness or had abused alcohol, drugs, or other substances; (15) had received long-term oral or intravenous glucocorticoids hormone therapy; or (16) had stress conditions such as surgery or severe trauma.

### Randomization and masking

The biostatistician, who did not participate in the enrolment of participants, used the statistical software SAS 8.2 PROC PLAN to generate a random code list for 300 participants to receive specific treatments. The stratified, blocked randomization method was used at each center. The test and placebo drugs were provided by the coordinating center in identical internal and external packaging. All of the randomized grouping number segments were sent to the research centers with the corresponding treatment drugs. The study was a double-blind, dual-simulation trial. Participants, investigators, and individuals involved in the analysis of trial data were masked to treatment assignments. A two-level blinding design was used, with the first level being by group (groups A-D) according to the case number, and the second level by treatment (berberine, *Bifidobacterium*, combination, and placebo).

### Procedures

The study included a run-in period of 2 weeks, in which diabetes education and lifestyle intervention were conducted, and a treatment period of 16 weeks. At the end of the run-in period, 300 eligible subjects were randomly assigned to the following four groups in a ratio of 1:2:1:2 for 16 weeks of treatment: berberine (Be), *Bifidobacterium* (Bi), berberine and *Bifidobacterium* (BB), and placebo group. The Be group received oral berberine tablets, 0.5 g, twice a day, and *Bifidobacterium* placebo capsules, twice a day. The Bi group received *Bifidobacterium* viable capsules (including 10^8^ *Bifidobacterium adolescentis*), 0.70 g, twice a day, and berberine placebo tablets, twice a day. The BB group received *Bifidobacterium* viable capsules, 0.70 g, twice a day, and berberine tablets, 0.5 g, twice a day. The placebo group received *Bifidobacterium* placebo capsules twice a day, and berberine placebo tablets, twice a day. The preparations used were berberine tablets (Northeast Pharmaceutical Group Shenyang First Pharmaceutical Co., Ltd., Lot No.: 130917), berberine tablet simulants with the main component of starch (Northeast Pharmaceutical Group Shenyang First Pharmaceutical Co., Ltd., Lot No.: 20150101), *Bifidobacterium* viable capsules (Livzon Pharmaceutical Group Inc., Lot No.: 20141013), and *Bifidobacterium* viable capsules simulants with the main components of lactose and magnesium stearate (Livzon Pharmaceutical Group Inc., Lot No.: 141001). The appearances and characteristics of simulants were similar to berberine tablets and *Bifidobacterium* viable capsules, respectively.

All of the participants returned to the center every 4 weeks for planned visits during the double-blind intervention periods, and the remaining pills or strips were taken back upon completion of the study and counted to assess adherence. Researchers emphasized the importance of diet and exercise to subjects at each treatment visit. The telephone interview was conducted 2 weeks after the first administration of the study drug to record the medication status and adverse reactions of every participant. The time course for participant registration, intervention, assessment, and follow-up is shown in the Standard Protocol Items: Recommendations for Interventional Trials (see Additional file 1: Table S1).

### Outcomes

The primary efficacy endpoint was the absolute value of FPG compared with baseline after 16 weeks of treatment in all of the participants, which were assessed by each center. The secondary efficacy endpoints were the changes compared with baseline in (1) 2-hr PPG; (2) glycosylated hemoglobin (HbA1c); (3) blood pressure; (4) lipid metabolism, including total cholesterol (TC), low-density lipoprotein cholesterol (LDL-C), high-density lipoprotein cholesterol (HDL-C), and triglycerides (TG); (5) body weight (BW) and BMI; (6) homeostasis model assessment (HOMA) index and insulin early-phase and late-phase secretion index; (7) intestinal glucagon-like peptide-1 (GLP-1); and (8) gut microbiota.

Safety assessments were based on monitoring the vital signs, BW and physical examination, laboratory data, 12-lead electrocardiogram, hypoglycemia, and other adverse events.

### Data collection

The methods for the collection of most endpoint measurements were shown in published study protocol [26], except for the gut microbiota.

Feces samples freshly collected from each participant were immediately frozen at − 20 °C, transported to the laboratory in an ice pack and stored at − 80 °C upon arrival. Bacterial DNA was extracted at Novogene Bioinformatics Technology Co., Ltd. using a Tiangen kit according to the manufacturer’s instructions. All samples were paired-end sequenced on the Illumina NovaSeq 6000 (insert size 350 bp, read length 151 bp) at the Novogene Bioinformatics Technology Co., Ltd.

### Sequence analysis

Adaptor and low-quality reads were discarded from the raw reads, and the remaining reads were filtered in order to eliminate human host DNA based on the human genome reference (hg19). Taxonomic profiling of the metagenomic samples was performed using MetaPhlAn2 [27], which uses a library of clade-specific markers to provide pan-microbial quantification at the species level. To obtain the functional profile, the high-quality reads were aligned to the updated gut microbiome gene catalog using SOAP2 (v2.22) with a threshold of more than 90% identity over 95% of the length. Sequence-based gene abundance profiling was performed as previously described [28]. Pathway enrichment analyses are based on KEGG annotation and the reporter score analysis [29].

### Statistical analysis

This was a pilot study of oral *Bifidobacterium*. The sample size was estimated using the NCSS PASS 11 (NCSS LLC, Kaysville, UT, USA) software. The sample size calculation was modeled after a previously described method [26]. Based on the decrease of the primary efficacy endpoint of FPG and the results of other studies, the final sample size for each group was determined to be 50 for the Be group, 100 for the Bi group, 50 for the BB group, and 100 for the placebo group, for a total sample size of 300.

The intention-to-treat analysis for efficacy endpoints included participants who received at least one dose of the trial drug and had at least one post-treatment data point, comprising the full analysis set. The per-protocol set included participants who adhered adequately to the assigned regimen including undergoing the trial drug treatment according to the protocol without any significant protocol deviation and completing all the evaluations of this study. The safety analyses were based on the safety set, which contained participants who received at least one dose of the trial drug and had at least one safety assessment. The study did not incorporate a planned midpoint analysis.

Efficacy analyses were performed based on the full analysis set. Changes in the primary and secondary efficacy endpoints were assessed using an analysis of covariance model that included terms for grouping, study center, and baseline value. The least squares (LS) mean and corresponding 95% confidence intervals (CIs) were presented for the changes in each group, and Dunnett LS mean differences (95% CI) were provided for the differences between the groups. Differences were considered to be statistically significant when the 95% CI did not include 0. Unless otherwise specified, the method of the last observation carried forward was used for efficacy analysis with missing values. Safety assessments were analyzed and adverse events aggregated. The continuous variables or frequency counts and percentages in safety and tolerability data were analyzed using descriptive statistics. The relative indices for hypoglycemic events and changes in weight were compared between the groups according to general principles.

All of the data were analyzed using the SAS 9.3 (SAS Institute Inc., Cary, NC, USA) software with pre-programmed algorithms. Quantitative indices recorded included mean ± standard deviation and median; qualitative or grade indices were recorded using a frequency distribution table. Two-sided tests were used in all cases, and *P* < 0.05 was considered to be statistically significant. Fisher’s exact probability test was used to compare the attrition rates between the groups.

Statistical analyses of the gut microbiota were made using the R software. The differential abundance of phyla, genera, and species was tested with the two-tailed Wilcoxon rank-sum tests, and *P* < 0.05 was considered to be statistically significant. All data were not corrected for multiple testing.

## Results

### Participant characteristics

**Fig. 1:**
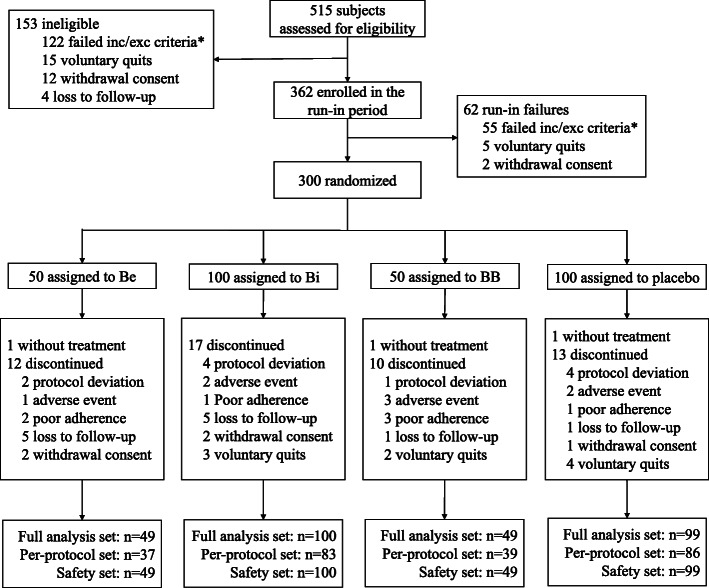
Trial profile. *The criteria were not mutually exclusive. The full analysis set, as the primary analysis set for this study, included participants who received at least one dose of the trial drug and had at least one post-treatment data point. The per-protocol set included participants who adhered adequately to the assigned regimen, including undergoing the trial drug treatment according to the protocol without any significant protocol deviation and completing all the evaluations of this study. The safety set included participants who received at least one dose of the trial drug and had at least one safety assessment

Between October 2015 and April 2018, 515 participants were screened. Three hundred of these participants were deemed to be eligible and completed randomization (Fig. 1). The intention-to-treat analysis was performed for a total of 297 participants of the full analysis set (Be group *n* = 49 participants, Bi group *n* = 100 participants, BB group *n* = 49 participants, and placebo group *n* = 99 participants). The per-protocol set included 245 participants. The safety set included 297 participants. Baseline demographic and clinical characteristics did not differ among the groups (Table 1).

**Table 1:** Baseline characteristics of subjects (full analysis set)

Variable	Be	Bi	BB	Placebo	***P*** value
*n* (%)	49 (98.00)	100 (100.00)	49 (98.00)	99 (99.00)	
Age, years	53.28 ± 9.87	54.16 ± 9.10	53.36 ± 9.49	52.73 ± 9.35	0.761
Sex (male), *n* (%)	23 (46.94)	59 (59.00)	27 (55.10)	54 (54.55)	0.585
BW, kg	68.37 ± 10.82	69.57 ± 9.06	69.04 ± 10.48	69.58 ± 11.24	0.904
BMI, kg/m^2^	25.05 ± 2.68	25.25 ± 2.28	25.20 ± 2.69	25.02 ± 2.80	0.921
Waist circumference, cm	90.08 ± 9.25	90.98 ± 7.58	91.82 ± 8.13	91.13 ± 8.58	0.774
Hip circumference, cm	98.17 ± 5.73	98.72 ± 7.74	98.60 ± 6.20	98.10 ± 6.81	0.920
Systolic blood pressure, mmHg	120.56 ± 11.88	123.08 ± 12.42	121.17 ± 13.75	122.71 ± 11.37	0.591
Diastolic blood pressure, mmHg	77.46 ± 8.94	78.57 ± 8.19	77.97 ± 7.82	76.86 ± 7.68	0.516
Pulse, beats/min	73.14 ± 8.29	74.15 ± 8.56	74.80 ± 7.20	74.17 ± 9.18	0.812
ECG					0.225
Normal, *n* (%)	39 (79.59)	64 (64.00)	29 (59.18)	63 (63.64)	
Non-clinical significance, *n* (%)	9 (18.37)	33 (33.00)	17 (34.69)	26 (26.26)	
Clinical significance, *n* (%)	1 (2.04)	3 (3.00)	3 (6.12)	9 (9.09)	
None, *n* (%)	0 (0.00)	0 (0.00)	0 (0.00)	1 (1.01)	
FPG, mmol/L	6.49 ± 0.64	6.40 ± 0.66	6.42 ± 0.77	6.43 ± 0.71	0.881
30 min post-plasma glucose, mmol/L	12.12 ± 1.63	11.93 ± 1.84	11.76 ± 2.09	11.94 ± 1.86	0.830
2-hr PPG, mmol/L	11.17 ± 2.41	11.40 ± 3.08	11.24 ± 2.37	11.62 ± 3.08	0.906
HbA1c, %	6.19 ± 0.53	6.19 ± 0.59	6.12 ± 0.72	6.23 ± 0.68	0.920
Fasting serum insulin, μIU/mL	9.87 (5.91–13.60)	10.52 (7.65–13.88)	9.56 (7.49–13.89)	9.78 (6.69–11.98)	0.430
30 min post-serum insulin, μIU/mL	39.16 (21.46–64.74)	33.00 (19.75–61.83)	34.30 (20.40–51.74)	32.98 (23.88–52.84)	0.962
2-hr post-serum insulin, μIU/mL	61.85 (40.88–94.43)	58.56 (40.50–84.36)	69.15 (46.19–88.76)	56.26 (36.35–79.12)	0.279
Fasting serum C peptide, ng/mL	1.46 (0.60–2.04)	1.58 (0.60–2.28)	1.38 (0.72–2.13)	1.47 (0.63–2.13)	0.995
30 min post-serum C peptide, ng/mL	3.29 (2.07–4.44)	2.67 (1.52–3.87)	2.76 (1.77–3.84)	2.79 (1.08–4.53)	0.711
2-hr post-serum C peptide, ng/mL	6.01 (3.09–8.92)	5.64 (2.16–8.74)	6.76 (3.39–8.35)	5.82 (2.40–8.32)	0.745
TG, mmol/L	2.12 (1.50–2.61)	1.71 (1.21–2.46)	1.66 (1.03–2.14)	1.83 (1.20–2.35)	0.253
TC, mmol/L	5.03 ± 1.15	4.76 ± 1.01	4.44 ± 0.96	4.72 ± 0.88	0.035
HDL-C, mmol/L	1.15 ± 0.30	1.19 ± 0.31	1.12 ± 0.27	1.15 ± 0.26	0.535
LDL-C, mmol/L	3.00 ± 0.88	2.85 ± 0.80	2.67 ± 0.84	2.81 ± 0.79	0.260
HOMA-IR	2.69 (1.76–3.41)	3.05 (1.88–4.08)	2.74 (2.16–3.95)	2.75 (1.89–3.45)	0.450
HOMA-β	69.66 (42.97–98.37)	71.98 (53.23–105.48)	68.00 (46.41–96.77)	63.53 (48.40–87.79)	0.832
Fasting GLP-1, pmol/L	2.38 (1.64–5.24)	2.00 (1.53–5.11)	2.95 (1.52–5.82)	2.71 (1.66–5.99)	0.895
30 min post-GLP-1, pmol/L	4.39 (2.71–7.03)	4.62 (2.62–7.29)	3.77 (2.62–7.28)	3.98 (2.37–6.97)	0.982
2-hr post-GLP-1, pmol/L	2.31 (1.24–4.34)	2.48 (1.80–5.04)	3.32 (1.92–5.32)	2.48 (1.40–5.04)	0.745
Area under the curve of GLP-1	7.48 (4.53–14.40)	8.13 (4.91–12.90)	6.99 (5.31–13.69)	6.95 (4.72–13.34)	0.905

Data were presented as *n* (%), mean ± standard deviation, and median (range interquartile). *P* value: comparison among the groups after treatment

ECG explanation: normal, normal ECG; non-clinical significance, abnormal ECG without any clinical significance; clinical significance, abnormal ECG with clinical significance; none, ECG was not performed

HOMA-IR: (fasting serum insulin × fasting plasma glucose)/22.5, homeostasis model assessment index for assessing insulin resistance; HOMA-β: (20 × fasting serum insulin)/(fasting plasma glucose − 3.5), homeostasis model assessment index for assessing β cell function. *P* value: comparison among the groups; Be: treatment with berberine; Bi: treatment with *Bifidobacterium*; BB: treatment with berberine and *Bifidobacterium*

*BW* body weight, *BMI* body mass index, *FPG* fasting plasma glucose, *2-hr PPG* 2-hour postprandial plasma glucose, *TG* triglyceride, *TC* total cholesterol, *HDL-C* high-density lipoprotein cholesterol, *LDL-C* low-density lipoprotein cholesterol, *ECG* electrocardiogram

### Changes in primary and secondary efficacy endpoints

The changes in the primary and secondary efficacy endpoints between baseline and after 16 weeks of treatment are shown in Table 2. The primary efficacy endpoint, FPG, changed from 6.49 ± 0.64 at baseline to 6.20 ± 0.80 mmol/L at 16 weeks in the Be group, from 6.40 ± 0.66 to 6.43 ± 0.93 mmol/L in the Bi group, from 6.42 ± 0.77 to 6.16 ± 0.91 mmol/L in the BB group, and from 6.43 ± 0.71 to 6.67 ± 1.34 mmol/L in the placebo group. Compared with the placebo group, a pronounced effect of lowering FPG was observed in the Be and BB groups with an LS mean difference of − 0.50, 95% CI [− 0.85, − 0.15] mmol/L and − 0.55, 95% CI [− 0.91, − 0.20] mmol/L, respectively. However, there was no significant difference between the Be and BB groups with an LS mean difference of − 0.05, 95% CI [− 0.47, 0.36] mmol/L. In addition, reduction of FPG was not shown in the Bi group (LS mean difference of − 0.19, 95% CI [− 0.47, 0.09] mmol/L).

**Table 2:** The primary and secondary endpoints after treatments (full analysis set)

Variable	Be		Bi		BB		Placebo		***P*** value	***P***_**change**_ value
	16 weeks	Change	16 weeks	Change	16 weeks	Change	16 weeks	Change		
**Primary efficacy endpoint**										
FBG, mmol/L	6.20 ± 0.80	− 0.37 (− 0.69, − 0.06)^a^	6.43 ± 0.93	− 0.06 (− 0.28, 0.16)^b^	6.16 ± 0.91	− 0.42 (− 0.73, − 0.12)^a^	6.67 ± 1.34	0.13 (− 0.09, 0.35)	0.067	0.004
**Secondary efficacy endpoint**										
2-hr PPG, mmol/L	9.84 ± 2.93	− 1.16 (− 2.09, − 0.23)^a^	11.33 ± 3.50	0.01 (− 0.64, 0.65)^b^	9.79 ± 2.49	− 1.37 (− 2.27, − 0.48)^a^	11.49 ± 3.66	0.21 (− 0.44, 0.87)	0.004	0.004
HbA1c, %	6.08 ± 0.52	− 0.05 (− 0.19, 0.08)	6.15 ± 0.61	0.02 (− 0.07, 0.12)^b^	5.90 ± 0.52	− 0.22 (− 0.36, − 0.09)^a^	6.14 ± 0.66	0.00 (− 0.10, 0.10)	0.111	0.016
TG, mmol/L	1.77 (1.19–2.47)	− 0.14 (− 0.46, 0.19)	1.81 (1.32–2.54)	0.04 (− 0.18, 0.27)	1.41 (0.99–2.05)	− 0.18 (− 0.50, 0.15)	1.73 (1.12–2.13)	− 0.06 (− 0.29, 0.17)	0.514	0.605
TC, mmol/L	4.64 ± 1.15	− 0.25 (− 0.50, 0.00)^a^	4.81 ± 1.03	0.02 (− 0.15, 0.19)^b^	4.14 ± 0.94	− 0.38 (− 0.63, − 0.13)^a^	4.79 ± 0.94	0.06 (− 0.12, 0.24)	0.002	0.004
HDL-C, mmol/L	1.14 ± 0.26	0.00 (− 0.06, 0.07)^a^	1.21 ± 0.33	0.04 (0.00, 0.09)^a^	1.15 ± 0.22	0.03 (− 0.03, 0.10)^a^	1.25 ± 0.31	0.11 (0.07, 0.16)	0.161	0.012
LDL-C, mmol/L	2.76 ± 0.81	− 0.15 (− 0.34, 0.05)	2.90 ± 0.81	− 0.00 (0.14, 0.14)^b^	2.41 ± 0.76	− 0.32 (− 0.52, − 0.12)^a^	2.85 ± 0.85	0.01 (− 0.13, 0.15)	0.011	0.018
BMI, kg/m^2^	24.47 ± 2.78	− 0.42 (− 0.70, − 0.14)	24.90 ± 2.19	− 0.37 (− 0.56, − 0.17)	24.96 ± 2.81	− 0.30 (− 0.57, − 0.03)	24.88 ± 2.61	− 0.19 (− 0.39, 0.01)	0.797	0.409
BW, kg	66.69 ± 10.12	− 1.29 (− 2.09, − 0.48)	68.39 ± 8.56	− 1.03 (− 1.59, − 0.46)	68.67 ± 10.74	− 0.93 (− 1.71, − 0.15)	69.31 ± 10.64	− 0.42 (− 0.99, 0.16)	0.576	0.204
Waist circumference, cm	86.64 ± 8.75	− 2.19 (− 3.41, − 0.96)	89.78 ± 7.40	− 1.26 (− 2.11, − 0.41)	90.50 ± 8.32	− 0.81 (− 2.00, 0.37)	90.22 ± 8.22	− 0.92 (− 1.79, − 0.05)	0.087	0.284
HOMA-IR	2.56 (1.87–3.36)	0.04 (− 0.62, 0.71)	2.70 (1.89–4.25)	− 0.10 (− 0.58, 0.38)	2.28 (1.78–3.02)	− 0.63 (− 1.29, 0.03)	2.72 (1.58–3.86)	− 0.12 (− 0.61, 0.36)	0.815	0.446
HOMA-β	68.8 (51.98–111.85)	6.52 (− 4.59, 17.62)	67.51 (49.80–101.45)	0.49 (− 7.56, 8.54)	69.03 (46.59–111.06)	4.42 (− 6.54, 15.38)	61.51 (43.24–95.42)	− 5.78 (− 13.87, 2.31)	0.313	0.215
Fasting GLP-1, pmol/L	2.26 (1.08–4.63)	1.36 (− 0.64, 3.36)	1.93 (1.27–3.89)	− 1.67 (− 3.10, − 0.25)	2.40 (1.34–3.68)	− 1.71 (− 3.66, 0.23)	2.23 (1.20–5.16)	− 0.87 (− 2.32, 0.57)	0.901	0.055
30 min post-GLP-1, pmol/L	3.17 (1.77–7.81)	− 0.70 (− 4.11, 2.71)	3.03 (1.98–6.11)	− 1.54 (− 3.96, 0.88)	3.18 (2.31–6.07)	− 0.72 (− 4.04, 2.59)	2.33 (1.54–5.73)	0.77 (− 1.67, 3.21)	0.572	0.529
2-hr post-GLP-1, pmol/L	3.17 (2.26–5.22)	0.94 (− 0.25, 2.12)	2.31 (1.93–4.10)	− 0.89 (− 1.73, − 0.05)	2.68 (1.83–3.91)	− 0.70 (− 1.86, 0.45)	2.78 (1.80–4.08)	− 0.40 (− 1.25, 0.44)	0.153	0.062
AUC GLP-1	6.43 (3.81–11.75)	− 0.25 (− 4.34, 3.83)	6.02 (4.05–9.67)	− 2.83 (− 5.75, 0.09)	5.70 (4.22–7.62)	− 2.31 (− 6.24, 1.62)	5.48 (3.68–11.54)	0.02 (–2.93, 2.97)	0.470	0.427

Data after treatment were presented as mean ± standard deviation and median (range interquartile); *P* value: comparison among the groups after treatment

Change: values were least-squares means represent changes from baseline (95%CI). *P*_change_ value: changes comparison among groups; Be: treatment with berberine; Bi: treatment with *Bifidobacterium*; BB: treatment with berberine and *Bifidobacterium*

*FPG* fasting plasma glucose, *2-hr PPG* 2-hour postprandial plasma glucose, *TG* triglyceride, *TC* total cholesterol, *HDL-C* high-density lipoprotein cholesterol, *LDL-C* low-density lipoprotein cholesterol, *BW* body weight, *BMI* body mass index

^a^Significant differences between the group and placebo group

^b^Significant differences between the group and BB group

We also found similar improvements of 2-hr PPG to FPG. Compared with the placebo group, more reductions of 2-hr PPG were observed in the Be and BB groups with an LS mean difference of − 1.37, 95% CI [− 2.42, − 0.32] mmol/L and − 1.59, 95% CI [− 2.62, − 0.55] mmol/L, respectively. Only the BB group showed a significant reduction of HbA1c compared with the placebo group with an LS mean difference of − 0.23, 95% CI [− 0.38, − 0.07] %, instead of the Be group (LS mean difference of − 0.06, 95% CI [− 0.21, 0.10] %) (Table 2), which may indicate that a better hypoglycemic effect was observed in the BB group. There were significant differences in lipid levels among the groups, including TC, LDL-C, and HDL-C. The similar effect of lowering TC level was shown in the Be group and BB group when compared with the placebo group, with an LS mean difference of − 0.31, 95% CI [− 0.59, − 0.04] mmol/L and − 0.44, 95% CI [− 0.72, − 0.16] mmol/L, respectively. The changes of TC level in the Bi group were similar to the placebo group (LS mean difference of − 0.04, 95% CI [− 0.26, 0.18] mmol/L). Only the BB group showed that LDL-C was significantly decreased compared with the placebo group, with an LS mean difference of − 0.32, 95% CI [− 0.55, − 0.10] mmol/L. The HDL-C levels were slightly increased in the Be, Bi, and BB groups compared with the placebo group, with an LS mean difference of − 0.11, 95% CI [− 0.18, − 0.03] mmol/L, − 0.07, 95% CI [− 0.13, − 0.01] mmol/L, and − 0.08, 95% CI [− 0.15, − 0.01], respectively. Additionally, there were no significant differences in other metabolic-related indicators among the groups.

We conducted sensitivity analyses based on per-protocol set, which showed similar results with those based on full analysis set (see Additional file 2: Table S2).

### Comparison of composition and function of gut microbiota

To characterize the gut microbiota of all groups, 208 high-quality, paired fecal samples collected at baseline and 16 weeks (Be group, *n* = 30; Bi group, *n* = 62; BB group, *n* = 30; and placebo group, *n* = 86) were performed with whole metagenomics sequencing. Next, we compared the demographic and clinical characteristics between participants with and without fecal samples, but there was no significant difference between them (see Additional file 3: Table S3). At the community level, more changes in the structure of the gut microbiota were observed in the Be and BB groups after treatment. The gene richness of the Be group decreased significantly compared with baseline (see Additional file 4: Fig. S1A, *P* = 0.009), and the BB group also showed decreased tendency (see Additional file 4: Fig. S1A, *P* = 0.055). The Bray-Curtis distance showed that gut microbiota changes between baseline and after treatment in the Be and BB groups were significantly larger than those in the Bi and placebo groups (see Additional file 4: Fig. S1B). There were no significant differences in gene richness or Bray-Curtis distance between the Bi and placebo groups.

**Fig. 2:**
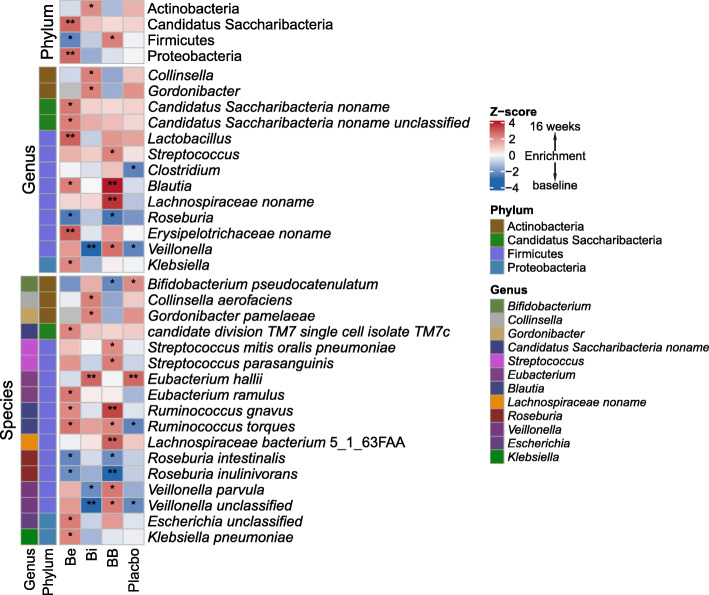
Heat map showing differentially abundant taxa of the fecal microbiota between baseline and 16 weeks of treatment in four experiments. The values of color in the heat map represent the *Z*-score. Only bacterial taxa that were significant in one of the experiments were included. The Wilcoxon matched-pairs signed-rank tests were used, *P < 0.05; **P < 0.01

After filtering out species with a low occurrence (i.e., present in fewer than 30% of individuals), we found that the abundance of Firmicutes was slightly lowered in the Be group, but higher in the BB group. Consistent changes were also observed in the Be group and BB group, including decreased abundance of *Roseburia* and increased abundance of *Blautia* including *Ruminococcus gnavus* and *Ruminococcus torques*. The abundance of Actinobacteria was increased only in the Bi group (Fig. 2). Furthermore, we found that the abundance of Proteobacteria was significantly higher in the Be group, including the opportunistic pathogen *Klebsiella pneumoniae*. However, this phenomenon was not observed in the BB group, which may be attributable to the use of *Bifidobacterium*.

According to further analysis about functional characterization, we found that some carbohydrate and lipid metabolism pathways, including galactose metabolism (map00052), fructose and mannose metabolism (map00051), and glycerolipid metabolism (map00561) pathways, were enriched in the Be and BB groups, which may mainly result from the use of berberine (see Additional file 5: Table S4). The Be group was characterized by enriched pathways of xenobiotic biodegradation and metabolism associated with harmful chemical products, including toluene degradation (map00623), benzoate degradation (map00362), fluorobenzoate degradation (map00364), and chlorocyclohexane and chlorobenzene degradation (map00361), but these changes were inverse in Bi group (see Additional file 5: Table S4). Whether this phenomenon was another benefit of *Bifidobacterium* needs to be further assessed.

### Safety assessments

There were no significant differences among the groups in the incidence of adverse events, any drug-related adverse events, any adverse events leading to discontinuation, and severe adverse events. Hypoglycemia occurred seven times in seven participants of the BB group, 14 times in nine participants of the Be group, 23 times in 14 participants of the Bi group, and 19 times in 14 participants of the placebo group. There were also no significant differences in the incidence of hypoglycemia among the groups (Table 3).

**Table 3:** Summary of adverse events (safety set)

Adverse events	Be (*n* = 49)		Bi (*n* = 100)		BB (*n* = 49)		Placebo (*n* = 99)		***P*** value
	Events, *n*	Participant, *n* (%)	Events, *n*	Participant, *n* (%)	Events, *n*	Participant, *n* (%)	Events, *n*	Participant, *n* (%)	
Adverse events	111	40 (81.63)	203	84 (84.00)	118	40 (81.63)	239	82 (82.83)	0.973
Drug-related adverse events	19	9 (18.37)	30	21 (21.00)	28	15 (30.61)	37	26 (26.26)	0.430
Adverse events unrelated to the study drug	92	38 (77.55)	173	81 (81.00)	90	37 (75.51)	202	79 (79.80)	0.859
Adverse events leading to discontinuation	4	2 (4.08)	4	4 (4.00)	0	0 (0.00)	6	3 (3.03)	0.679
Severe adverse events	5	4 (8.16)	4	4 (4.00)	0	0 (0.00)	6	3 (3.03)	0.213
Hypoglycemia event	14	9 (18.37)	23	14 (14.00)	7	7 (14.29)	19	14 (14.14)	0.887
Most frequent treat-related adverse events									
Fecal abnormalities	5	3 (6.12)	12	8 (8.00)	7	6 (12.24)	12	9 (9.09)	
Abdominal discomfort/digestive tract disease	9	5 (10.20)	7	6 (6.00)	14	9 (18.37)	8	7 (7.07)	
Dental and oral disorders	0	0 (0.00)	0	0 (0.00)	1	1 (2.04)	1	1 (1.01)	
Others	1	1 (2.04)	1	1 (1.00)	0	0 (0.00)	0	0 (0.00)	
Upper respiratory tract infection	0	0 (0.00)	0	0 (0.00)	2	2 (4.08)	1	1 (1.01)	
Changes in body weight	0	0 (0.00)	3	3 (3.00)	1	1 (2.04)	1	1 (1.01)	
Dizziness	0	0 (0.00)	1	1 (1.00)	2	2 (4.08)	1	1 (1.01)	
Trauma/arthropathy	0	0 (0.00)	1	1 (1.00)	0	0 (0.00)	0	0 (0.00)	
Abnormal ECG/cardiac dysfunction	3	3 (6.12)	1	1 (1.00)	1	1 (2.04)	2	2 (2.02)	
Blood routine/biochemistry/urinalysis	1	1 (2.04)	4	3 (3.00)	0	0 (0.00)	10	7 (7.07)	
Hemorrhoids	0	0 (0.00)	0	0 (0.00)	0	0 (0.00)	1	1 (1.01)	

## Discussion

This study has shown that FPG were decreased significantly after 16 weeks of treatment in both the Be and BB groups. However, lowering 2-hr PPG showed a stronger effect in the BB group. No obvious hypoglycemic effect was observed in the Bi group. We also characterized the gut microbiota of all the groups and found that berberine could regulate the gut microbiota in human hyperglycemia, which provided some evidence for the intestinal mechanism underlying berberine’s hypoglycemic effect. To the best of our knowledge, this was the first multi-center, randomized, parallel-controlled study to report the hypoglycemic effect of berberine and single *Bifidobacterium* that were used synergistically on patients with hyperglycemia.

As expected, our results showed that berberine (0.5 g, twice a day) had a hypoglycemic effect in patients newly diagnosed with hyperglycemia compared with placebo, especially in reducing FPG and 2-hr PPG. This was consistent with the results from previous clinical trials [30, 31], which indicated that berberine treatment was associated with different degrees of reduction on FPG, 2-hr PPG and HbA1c. However, significant changes in HbA1c were not observed in the Be group. This may be attributed to the relatively low mean glucose level of enrolled participants, or perhaps the treatment period was not long enough. Therefore, this study provided evidence for the hypoglycemic effect of berberine in the relatively early stage of T2D.

Moreover, although more effective lowering of the HbA1c effect was not observed directly in the BB group compared with the Be group (LS mean difference − 0.17, 95% CI [− 0.35, 0.01] %), only the BB group showed a significant reduction of HbA1c compared with the placebo group (LS mean difference of − 0.23, 95% CI [− 0.38, − 0.07] %), instead of the Be group (LS mean difference of − 0.06, 95% CI [− 0.21, 0.10] %). These results indicated that *B. adolescentis* may enhance the hypoglycemic effect of berberine. Supplementation with *B. adolescentis* in a rodent model of the metabolic syndrome has been shown to increase insulin sensitivity [32]. *Bifidobacterium* viable capsules (*B. adolescentis*) used in this trial are approved by the Chinese State Food and Drug Administration for clinical use (approval number S10960040) to treat intestinal dysfunction caused by an imbalance of the gut microbiota in clinical practice. The detailed drug instruction can be found on the website (https://www.livzon.com.cn/product/21.html). Patients with T2D were characterized by a moderate degree of gut microbial dysbiosis [33]. Furthermore, a study reported that metformin promoted the growth of *B. adolescentis* both in vivo and in vitro using pure cultures. The study also observed a negative correlation between the peak-to-trough ratio of *B. adolescentis* and HbA1c, which suggested that increased growth of this bacterial species could potentially contribute to the antidiabetic effect of metformin [4]. Metformin and berberine similarly shifted the overall structure of the gut microbiota in rats [34]. Therefore, *B. adolescentis* may improve the intestinal environment to promote berberine’s ability to play a hypoglycemic role. Unfortunately, the hypoglycemic effect of *B. adolescentis* was not observed in our study. Another study also showed that there was not a superior effect of probiotics (multi-strain probiotics, ≥ 50 billion CFU) in treating T2D compared to that of placebo or probiotics plus berberine or compared to that of berberine [31]. The types and dosages of probiotics used were also different from this study. We chose the dose of 2 × 10^8^ CFU per day according to the manufacturer’s drug instructions for safety reasons. The low dose of probiotics in this study should be acknowledged. The optimal and most clinically relevant probiotic medium, strain, dose, and duration of intervention have not yet been fully described. Data in a review suggested that multi-strain probiotic interventions providing seven million to 100 billion CFU administered for 6 to 12 weeks are efficacious for improving glycemic control in T2D patients [35]. Therefore, whether the different probiotic strains, the number of probiotics ingested, or the patient’s race could lead to different hypoglycemic effects are worthy topics of further study.

From animal and in vitro studies, berberine is known to act as an antidiabetic agent through stimulating the glucose uptake of cells, inhibiting gluconeogenesis to reduce hepatic glucose output, increasing the expression of insulin receptors and the secretion of GLP-1, and suppressing the activity of intestinal disaccharidases [36]. A variety of molecular mechanisms for berberine have been proposed, such as AMPK activation, glucose transport stimulation, mitochondrial inhibition, and anti-oxidation [36]. Moreover, berberine could regulate the gut microbiota, increase the production of gut short-chain fatty acids, alter microbial bile acid metabolism and the intestinal farnesoid X receptor signaling pathway, and affect the synthesis and transport of amino acids [10]. To discern the intestinal mechanism of berberine’s hypoglycemic effect, we tested fecal samples of participants and found that the gut microbiota after treatment changed more compared with baseline in the BB and Be groups. The significant colonization of *B. adolescentis* was not observed after oral supplementation with *B. adolescentis*, yet probiotic gut mucosal colonization efficacy remains controversial [37]. Further study will be needed to decide whether this phenomenon occurs on account of low-dose probiotics or high levels of acid and bile acids in the stomach and duodenum. In addition, significant increases of *Blautia* were observed in both the BB and Be groups. *R. gnavus* and *R. torques* belonging to *Blautia* were consistently enriched in both the Be and BB groups compared with baseline. Indeed, *R. gnavus* was significantly correlated with the improvement of glucose homeostasis and insulin sensitivity [38]. *R. gnavus* has been reported to be an ursodeoxycholic acid (UDCA) producer, and its colonization was uniquely associated with the production of UDCA in children [39, 40]. The pharmaceutical benefits of UDCA are well known, as it is commonly administrated for the treatment or prevention of various diseases or symptoms associated with disorders of bile acid metabolism [41]. *R. gnavus* may be involved in intestinal tryptophan metabolism [42] and excretion of the neurotransmitter tryptamine in vitro [43]. It is also reported that *R. gnavus* expresses α-galactosidase, which plays essential roles in the metabolism of dietary oligosaccharides [44] and thus may be associated with galactose metabolism enrichment in the BB and Be groups in functional analysis. Therefore, whether *R. gnavus* potentiates the hypoglycemic effect of berberine must be further verified by animal experiments.

However, not all of the changes observed in the regulatory effect of berberine on patients with T2D were beneficial, including a decrease of some probiotics (such as the butyric acid producer *Roseburia*) and an increase of some opportunistic pathogens (Proteobacteria and *Streptococcus*); this observation is consistent with other studies [9, 12]. However, the increase of opportunistic pathogen growth caused by berberine could be offset through the supplementation of *Bifidobacterium*. The species of *Klebsiella pneumoniae*, *Roseburia intestinalis*, *Roseburia hominis*, and *R. gnavus* were regarded as the key berberine-responding species in another study [31]. They also found that the hypoglycemic effect of berberine was mediated by the gut microbiota, which inhibited the biotransformation of deoxycholic acid [31]. However, it was different from our study in that participants were given an oral broad-spectrum antibiotic for 7 days before berberine treatment [31].

We also observed significant decreases in gene richness in the Be group and mild decreases in the BB group, which may be correlated to the antibacterial activity by berberine, and decreased gene richness is mildly reversed by *Bifidobacterium*. Li et al. showed that the concomitant use of prebiotic could slightly reverse the reduced diversity and richness of microbiota caused by berberine and produce better glycometabolism than berberine alone in diabetic mice [45]. Another possible benefit of *Bifidobacterium* was observed in the function analysis. It could also offset enriched pathways of harmful chemical products by berberine including toluene, benzoate, fluorobenzoate, chlorocyclohexane, and chlorobenzene degradation. We also found some carbohydrate metabolism pathways, which were associated with the production of short-chain fatty acids. These were enriched in groups with berberine including galactose, fructose, and mannose metabolism [46]. Another study also showed that berberine could increase the intestinal short-chain fatty acids content in db/db mice [47].

Berberine could affect the secretion of GLP-1 in vivo and in vitro directly or indirectly [48, 49]. However, the evidence that berberine regulates human GLP-1 secretion is still absent. Although our results also showed that changes in GLP-1 concentration were similar among the groups, one cannot rule out that the lack of change in plasma GLP-1 concentration is not affected by berberine or *Bifidobacterium*. In fact, GLP-1 is secreted after a meal in the portal vein, where it exerts its physiologic role [50] and is rapidly degraded by the DPP-IV enzyme within 90 s [51]. Therefore, further study is needed to determine whether berberine increases GLP-1 secretion in humans.

Some changes in gut microbiota composition and functional analysis were also observed in the placebo and Bi groups. This result could indicate that some functional changes may be related to dietary (and exercise) recommendations in the period. Lifestyle intervention was shown to modify the gut microbiota in metabolic diseases [52, 53]. We observed that *Bifidobacterium pseudocatenulatum* and *Eubacterium hallii* were increased after intervention in the placebo group. *E. hallii* was also significantly increased in the Bi group. *E. hallii* was significantly correlated with the improvements of glucose homeostasis and insulin sensitivity [38]. Oral administration of *B. pseudocatenulatum* could reverse the adverse effects of diet-induced obesity through the gut-bone axis [54]. Both the Bi and placebo groups showed that the pathways of glycosaminoglycan degradation were increased and benzoate degradation was decreased. The functional analysis also showed some beneficial changes, including decreased pathways of ABC transporters and lipopolysaccharide biosynthesis after intervention in the placebo group.

Nonetheless, some limitations of this study should be acknowledged. First, this multi-center trial was conducted in the same geographic area; therefore, the conclusions may not be generalizable to other regions. Whether this phenomenon is prevalent across multiple regions needs further verification. Second, we observed the hypoglycemic effect at 16 weeks, but it is unknown whether prolonged treatment will cause further changes to the gut microbiota and other metabolic indices. Third, the sample size of stool was limited for subgroup analysis. Fourth, detailed dietary records were absent. Finally, further studies in vitro and on animals were not performed to explain the potential mechanism.

## Conclusions

In summary, the hypoglycemic effect of berberine was further validated, and *Bifidobacterium* showed the potential to enhance the hypoglycemic effect of berberine. We also observed possible changes in the gut microbiota when regulated by berberine for lowering glucose, which provided a basis for the study of its hypoglycemic mechanism and for clinical use of berberine as a safe and effective hypoglycemic drug in human hyperglycemia. Given the benefits of *Bifidobacterium* and berberine lowering blood glucose, more studies are needed to confirm these findings.

## Supplementary Information

**Additional file 1: Table S1.** Specific research plan and implementation steps.

**Additional file 2: Table S2.** The primary and secondary endpoints after treatments (per-protocol set).

**Additional file 3: Table S3.** Comparison of demographic characteristics between subjects with and without fecal samples.

**Additional file 4: Figure S1.** Differences in four groups on microbial community diversity between baseline and 16 weeks.

**Additional file 5: Table S4.** Reporter score of the KEGG pathways for comparisons between baseline and treatment.

## Data Availability

Sequence data for all microbiome samples are available in the European Nucleotide Archive repository with the accession number PRJEB44907 (https://www.ebi.ac.uk/ena/browser/view/PRJEB44907) [55]. The demographic and clinical dataset of the trial is not available due to this is not compliant with the ethical approval obtained for the study, but can be obtained from the corresponding authors upon reasonable request.

## Abbreviations

T2D: Type 2 diabetes
FPG: Fasting plasma glucose
2-hr PPG: 2-hour postprandial plasma glucose
BMI: Body mass index
TC: Total cholesterol
LDL-C: Low-density lipoprotein cholesterol
HDL-C: High-density lipoprotein cholesterol
LS: Least squares
CI: Confidence interval
UDCA: Ursodeoxycholic acid
