# Supplementary material for: Effectiveness and safety of Bifidobacterium and berberine in human hyperglycemia and their regulatory effect on the gut microbiota: a multi-center, double-blind, randomized, parallel-controlled study
Source: Genome Med. 2021 Aug 9;13:125. doi: 10.1186/s13073-021-00942-7 (PMC8351344; doi:10.1186/s13073-021-00942-7)
Supplement: Supplementary file 4 — Additional file 4: Figure S1. Differences in four groups on microbial community diversity between baseline and 16 weeks. [file 13073_2021_942_MOESM4_ESM.pdf]

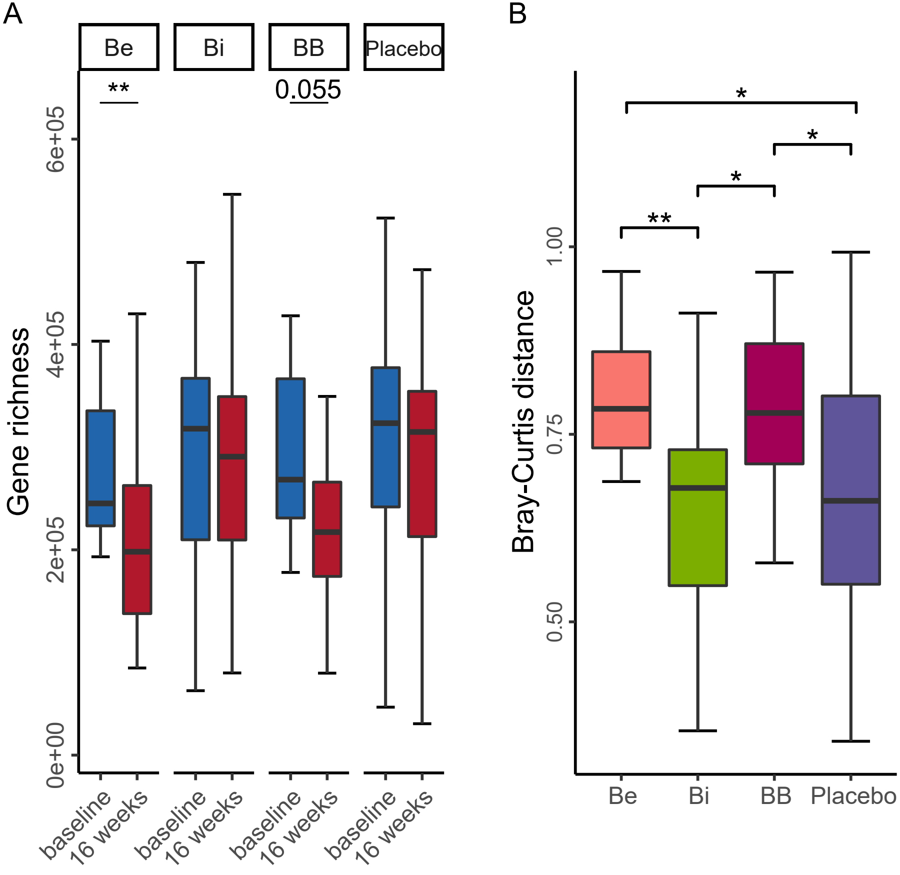

A
Be
Bi
BB
Placebo
**
0.055
Gene richness
6e+05
4e+05
2e+05
0e+00
baseline
16 weeks
baseline
16 weeks
baseline
16 weeks
baseline
16 weeks
B
Bray-Curtis distance
1.00
0.75
0.50
*
*
*
**
Be
Bi
BB
Placebo

Fig. S1: Differences in four groups on microbial community diversity between baseline and 16 weeks.

(A) Gene richness between baseline and 16 weeks in four experiments. Wilcoxon matched-pairs signed rank test, ** $P<0.01$. (B) Microbiota changes between baseline and 16 weeks for every subject in four experiments, Bray-Curtis distance were used. Wilcoxon rank-sum tests, ** $P<0.01$, * $P<0.05$.
